# Bacterial Colonisation: From Airborne Dispersal to Integration Within the Soil Community

**DOI:** 10.3389/fmicb.2022.782789

**Published:** 2022-05-09

**Authors:** Lucie A. Malard, David A. Pearce

**Affiliations:** ^1^Department of Applied Sciences, Faculty of Health and Life Sciences, Northumbria University, Newcastle-upon-Tyne, United Kingdom; ^2^Department of Ecology and Evolution, University of Lausanne, Lausanne, Switzerland; ^3^British Antarctic Survey, Natural Environment Research Council, Cambridge, United Kingdom

**Keywords:** Arctic ecosystems, airborne dispersal, microbial colonisation, bacterial diversity, snow, soil

## Abstract

The deposition of airborne microorganisms into new ecosystems is the first stage of colonisation. However, how and under what circumstances deposited microorganisms might successfully colonise a new environment is still unclear. Using the Arctic snowpack as a model system, we investigated the colonisation potential of snow-derived bacteria deposited onto Arctic soils during and after snowmelt using laboratory-based microcosm experiments to mimic realistic environmental conditions. We tested different melting rate scenarios to evaluate the influence of increased precipitation as well as the influence of soil pH on the composition of bacterial communities and on the colonisation potential. We observed several candidate colonisations in all experiments; with a higher number of potentially successful colonisations in acidoneutral soils, at the average snowmelt rate measured in the Arctic. While the higher melt rate increased the total number of potentially invading bacteria, it did not promote colonisation (snow ASVs identified in the soil across multiple sampling days and still present on the last day). Instead, most potential colonists were not identified by the end of the experiments. On the other hand, soil pH appeared as a determinant factor impacting invasion and subsequent colonisation. In acidic and alkaline soils, bacterial persistence with time was lower than in acidoneutral soils, as was the number of potentially successful colonisations. This study demonstrated the occurrence of potentially successful colonisations of soil by invading bacteria. It suggests that local soil properties might have a greater influence on the colonisation outcome than increased precipitation or ecosystem disturbance.

## Introduction

Global dispersal of microorganisms has primarily been shown to occur through airborne transport *via* the aerosolisation of particles (Smets et al., 2016). Once airborne, microorganisms can travel thousands of kilometres (Smith et al., 2013; Barberán et al., 2014, 2015; Maki et al., 2019) and be deposited into the most remote places on Earth, from the Arctic (Harding et al., 2011; Cuthbertson et al., 2017; Šantl-Temkiv et al., 2018), to Antarctica (Pearce et al., 2009, 2010; Bottos et al., 2014; Archer et al., 2019). While microorganisms were once considered to be ubiquitously distributed with limitless dispersal capability (De Wit and Bouvier, 2006), recent studies have refuted this hypothesis and shown that ecological drift and dispersal limitation significantly influence the distribution of microorganisms (Fierer and Jackson, 2006; Bahram et al., 2018; Delgado-Baquerizo et al., 2018; Malard et al., 2019a). Thus, airborne microorganisms have the potential to invade and colonise all ecosystems across the globe.

The first step of microbial colonisation is the deposition within the environment itself (Mallon et al., 2015a; Kinnunen et al., 2016). This deposition process is generally categorised as ‘wet’ or ‘dry’ deposition. Dry deposition occurs following adherence to buildings, plants, water or soil surfaces, while wet deposition is caused by precipitation events, such as rain or snow (Smets et al., 2016; Reche et al., 2018). While the constant deposition of microorganisms into new environments is accepted and has been demonstrated (Peter et al., 2014; Weil et al., 2017; Reche et al., 2018), whether the deposited microorganisms become established and colonise the new environment long term is still a subject of debate. For example, Fløistrup et al. (2018) demonstrated the quick colonisation of sterile soils by airborne microorganisms while Evans et al. (2019) suggested that dispersal *via* rainfall altered the soil microbial response to drought without actively demonstrating microbial colonisation.

The Arctic snowpack is an ideal model with which to study colonisation following wet deposition (by snowfall) since it covers the Arctic tundra for 8–10 months of the year while also isolating the soil from outside influence (Winther et al., 2003). The snowpack acts as an ephemeral transition ecosystem for airborne microorganisms. Indeed, although the snowpack is seeded by airborne microorganisms, its own unique microbial community develops over time (Harding et al., 2011; Larose et al., 2013; Els et al., 2019; Malard et al., 2019b). The snowpack community is well adapted to surrounding environmental conditions (Russell, 1990; Margesin and Miteva, 2011; Maccario et al., 2015), one of the key factors in successful colonisation (Mallon et al., 2015a; Kinnunen et al., 2016). Once the snow starts to melt, the run-off travels vertically under gravity on flat terrain, to reach the frozen soil layer and infiltrate the soil (Gray et al., 2001; Iwata et al., 2008; Larose et al., 2013). However, the percolation of meltwater is a complex process influenced by soil properties, such as temperature, moisture or the presence of ice, but also by the rate of snowmelt (Gray et al., 2001; Iwata et al., 2008). The snowpack is a major source of potential colonists as it supports between 10^1^ and 10^4^ microbial cells per mL (Amato et al., 2007; Zhang et al., 2010; Hauptmann et al., 2014; Cameron et al., 2015). Snowmelt also creates a peak in nutrient and solute availability in soils (Lipson and Schmidt, 2004; Edwards et al., 2006; Buckeridge and Grogan, 2010; Larose et al., 2013). This resource pulse may facilitate the colonisation of soils by snow microorganisms (Mallon et al., 2015b). Furthermore, ecosystem disturbance, such as the sudden addition of water (Fierer et al., 2003; Orwin et al., 2016), may promote successful colonisation (Liu et al., 2012; De Roy et al., 2013). Therefore, snowmelt may be an opportunity for snow microorganisms to establish in rich and diverse soil communities, which would otherwise be difficult to colonise (Jousset et al., 2011). Generally, studies of microbial invasion and colonisation investigate a single (or very few) invader taxa inoculated at a high density into manipulated communities (van Elsas et al., 2012; Acosta et al., 2015; Mallon et al., 2015b, 2018; Fløistrup et al., 2018; Li et al., 2019). In this study, we investigated changes in soil bacterial communities during snowmelt and evaluated the colonisation potential of snow bacteria deposited into Arctic soils during and after snowmelt using microcosm experiments. In order to examine these changes in a realistic scenario, the invading (snow) and invaded (soil) communities were not manipulated.

The first set of experiments evaluated the influence of increased precipitation on the colonisation potential by using different melt rate scenarios. We simulated a seasonal average and a fast-saturating snowmelt rate to test the hypothesis that increased colonisation would be observed in the soils influenced by a fast flow rate due to a higher number of microorganisms deposited as well as increased ecosystem disturbance (analogue to increased precipitation). Indeed, under the current climate trend, precipitation (either rain or snowfall) is expected to increase in the region (Bintanja and Selten, 2014; Bintanja and Andry, 2017), and the resulting deeper snowpack may melt faster due to higher temperatures (Wipf and Rixen, 2010). Whether such an increase in microorganisms and available water resulting from a deeper snowpack will influence the colonisation success remains to be determined.

The second set of experiments investigated the colonisation potential of microorganisms deposited into soil ecosystems with different pH ranges. In Arctic soils, as elsewhere, pH is the primary driver of microbial community structure and diversity (Fierer and Jackson, 2006; Männistö et al. 2007; Chu et al., 2010; Malard and Pearce, 2018; Malard et al., 2019a), demonstrating that climatic conditions are not the only abiotic factor invaders have to adapt to. Soil pH in the Arctic region is primarily acidoneutral, although the full pH spectrum can be found locally (Hengl et al., 2017; Malard et al., 2019a). We evaluated the influence of soil pH on the colonisation potential during average snowmelt rates to test the hypothesis that increased colonisation would be observed in acidoneutral soils compared to acidic and alkaline soils. In many ways, acidic and alkaline soils are considered harsh environments requiring a wide range of adaptations (Glenn and Dilworth, 1991; Horikoshi, 1999; Torsvik and Øvreås, 2008). Although microorganisms within the snow can be expected to be well adapted to climatic conditions (Russell, 1990; Margesin and Miteva, 2011; Maccario et al., 2015), whether they have the capacity to adapt to harsh physicochemical properties or to fast changing environmental conditions to colonise soils remains unknown.

## Materials and Methods

### Approach

**Figure 1:**
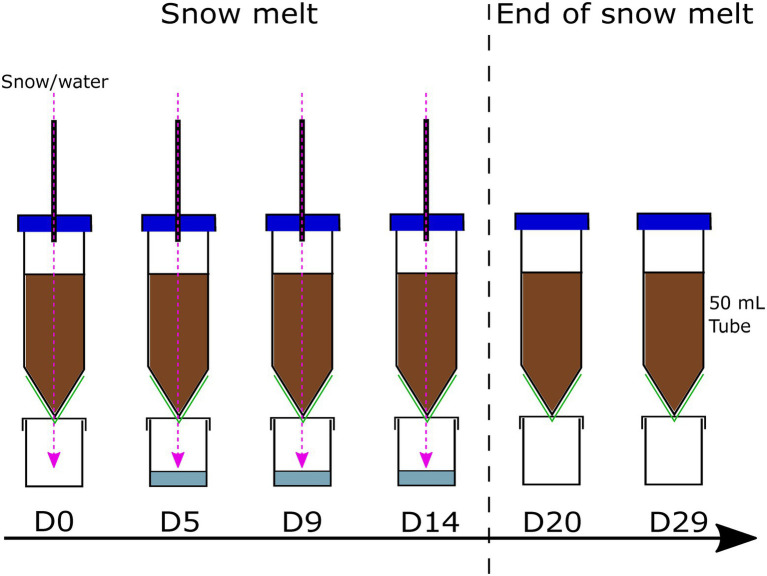
Microcosm construction. The snow/sterile water was input using a programmable peristaltic dosing pump, through a hole in the centrifugal tube cap. To allow infiltration and evacuation of the excess liquid, a hole was made at the bottom of the tube, covered with a membrane (green) allowing the easy passage of liquid and cells while retaining the soil in the microcosm system. Snow/sterile water was input every day for 14 days and stopped thereafter; the hole in the centrifugal tube cap was covered with autoclaved aluminium to avoid contamination from outside the system.

In all experiments, melted snow was input into soil columns and after percolation, the outflow was recovered (Figure 1). In the first set of experiments, only flow rate was manipulated (at acidoneutral soil pH) to evaluate the influence of increased precipitation (using snowmelt rate as a proxy). In the second set of experiments, only soil pH was manipulated (at constant average flow rate) to evaluate the influence of soil pH on the colonisation potential of snow bacteria into soils.

The snowmelt period in the Arctic lasts anywhere between 7 and 30 days, with an average of 15–20 days (Foster, 1989; Winther et al., 2002). Here, melted snow was introduced to the system every day for 14 days to simulate this snowmelt period, followed by 15 days snow-free to simulate the post-melt season. All experiments were conducted at 4°C to simulate Arctic climatic conditions. However, they were kept in the dark, unlike summer Arctic conditions, to avoid the formation of cyanobacterial mats and to avoid a light regime change after storage in the dark. Indeed, differentiating the impact of light and water regime change would have required a different set of experiments. Bacterial communities within each component (snow, soil, outflow) were monitored with time using qPCR and 16S rRNA gene amplicon sequencing.

### Sample Collection and Properties

The soil used for microcosm experiments was collected in Adventdalen (78°10′12″N, 16°3′0″E), Svalbard in July 2018, from the top 15 cm using ethanol-cleaned trowels and Whirl-pak bags (Nasco, WI, United States). The average temperature in July in Longyearbyen is 4°C. The soil was passed through a 1.5 cm sterilised sieve in a class II biological safety cabinet (ESCO, Singapore) to remove large organic matter and larger soil particles. pH, conductivity, moisture and total organic carbon were measured in the laboratory (Supplementary Table S1) using the same methods as Malard et al. (2019a). To avoid freezing the samples and the associated potential cell death (Soulides and Allison, 1961; Yanai et al., 2004), and because temperature was a constant factor in the microcosms, all materials were stored in the dark at 4°C after field sampling (DS) and for 100 days until the beginning of the experiments and were maintained at 4°C during the experiments. On the day of field sampling, 1 g of soil was frozen at −20°C for DNA extraction and later referred to as the day of field sampling (DS).

The snow used for microcosms was collected in Whirl-pak bags (Nasco) using ethanol-cleaned shovels, close to Mine 7 in Svalbard in July 2018. The snow was left to melt at room temperature, transferred to sterile containers and preserved at 4°C in the dark until transportation to the United Kingdom. On the day of field sampling (DS), 250 ml of melted snow was filtered through a 0.22 μm Whatman nitrocellulose filter (Merck, Darmstadt, Germany) using an ethanol-cleaned filtration unit (Nalgene Nunc International Corporation) and frozen at −20°C.

### Microcosm Construction

Prior to constructing the microcosms, a soil sample was frozen at −20°C and 250 ml of melted snow was filtered (using the same protocol as for DS) for DNA extraction, referred to as day 10 (D-10). Microcosms were constructed aseptically in a class II biological safety cabinet (ESCO) by adding 30 g of sieved soil in sterile 50 ml conical centrifugal tubes, packed at a density of approximately 0.96 g/cm^3^, within the range of expected density relating to the soil organic carbon in Arctic soils (Hossain et al., 2015). Microcosms were left for 10 days to allow the soil bacterial communities acclimatise and adapt to the environmental conditions until the start of the experiment (D0). A multichannel peristaltic dosing pump (Jebao DP-4, DP-5 and DP-3S) was used for the delivery of melted snow (treatment) and sterile water (controls) into each microcosm. At the bottom of each tube, an evacuation hole was created using a sterile needle to let the excess liquid exit the system and avoid complete water saturation of the microcosm. A 0.45 μm Durapore membrane filter (Merck) was fitted at the bottom of the tube to let the water and microorganisms pass while stopping the soil from leaving the microcosm. The outflow, referred to as flow-through (FT), was collected in a sterile container, replaced on each sampling day (Figure 1). Triplicates of each experiment were run in parallel for a total of 12 microcosms per experiment. Soil samples were collected aseptically from the top of the column using sterile spatulas on day 0 (D0, first day of the experiment), day 5 (D5), day 9 (D9), day 14 (D14, last day of melted snow input), day 20 (D20) and day 29 (D29). The collected soil was frozen at −20°C until further processing. On each sampling day, 250 ml of melted snow was filtered (using the same protocol as for DS) and frozen at −20°C until further processing. For control microcosms, sterile, filtered MilliQ water was added to a clean and empty autoclaved container and autoclaved again. The sterility of the water was assessed on each sampling day by microscopy using Petroff-Hausser chambers and DNA extractions (Supplementary Methods). The flow-through output was filtered through a 0.22 μm nitrocellulose filter (Merck) on D5, D9 and D14 and frozen at −20°C until further processing.

On each sampling day, the pH of input snow/water and soil columns was measured using a 5 ml of snow/water or a 1:5 soil to water ratio and a Mettler-Toledo FE20 pH meter (Mettler-Toledo Instruments Co., Shanghai, China). The input snow pH (6.33 ± 0.15) and sterile water pH (5.82 ± 0.36) were acidoneutral. Overall, each microcosm remained within the pH category assigned throughout the duration of the experiments (Supplementary Figure S1).

#### Snowmelt Rate Experiments

In the Arctic, snow melts at an average rate of 9 mm water equivalent (we) per day (Marsh and Woo, 1984; Foster, 1989; Winther et al., 2002, 2003). This average flow rate (9 mm we/day) was equivalent to 6.4 ml/day for a 50 ml centrifugal tube, which formed the basis of the microcosms. In Svalbard, in cases of extremely rapid melt, rates up to 68 mm we/day have been recorded (summarised in Winther et al., 2002). At this fast rate, pilot experiments demonstrated the rapid saturation of the system, preventing the percolation of water through the soil. To simulate a fast melt rate at which the input water would saturate and percolate, a melting rate of 35 mm we/day was selected (equivalent to 24.7 ml/day for a 50 ml centrifugal tube). Using the peristaltic pump, a volume of 6.4 ml was introduced once a day for average rates and a volume of 6.2 ml was introduced four times a day, every 3 h (for 10 h) for the fast rate. Triplicates of each experiment were run as follows: average rates with melted snow (treatment 1), average rates with sterile water (control 1), fast rates with melted snow (treatment 2) and fast rates with sterile water (control 2).

#### Soil pH Experiments

On the day of microcosm set-up (D-10), the pH of the already acidoneutral soil (Supplementary Table S1) was adjusted to cover acidic and alkaline pH ranges. Around 0.35 g of aluminium sulphate [Al_2_(SO_4_)_3_] was added to decrease soil pH and 0.44 g of calcium carbonate (CaCO_3_) was added to increase soil pH. The optimum mass of each chemical to add to the soil was tested in a pilot experiment and in accordance with Nicol et al. (2008). A total of 12 microcosms were prepared, six acidic with pH = 3.65 ± 0.14 and six alkaline with pH = 7.97 ± 0.22 and left for 10 days at 4°C to let the soil bacterial communities acclimatise and adapt to the new environmental conditions. Using the average melt rate of 9 mm we/day, triplicates of each experiment were as follows: acidic soil with melted snow (treatment 1), acidic soil with sterile water (control 1), alkaline soil with melted snow (treatment 2) and alkaline soil with sterile water (control 2). The acidoneutral microcosms (pH = 5.31 ± 0.17) with average melt rate were run in the previous experiment (melt rate) and not repeated. Instead, the results were reused in different settings to compare the experiments.

### DNA Extraction, Amplicon Sequencing, and Bioinformatic Processing

Soil DNA was extracted using the PowerSoil kit (Qiagen, Carlsbad, CA, United States) and following the manufacturers’ protocol. Snow and flow-through (FT) DNA was extracted using the PowerWater kit (Qiagen) and following the manufacturers’ protocol. Each extract was PCR amplified using the universal primers 515F-806R (Caporaso et al., 2010). Resulting amplicons were cleaned, normalised, pooled, sequenced on the Illumina MiSeq (as described in Malard et al., 2019a) and resulting amplicons were processed using the DADA2 pipeline v1.22 (Callahan et al., 2016). Forward and reverse read pairs were trimmed and filtered, with forward reads truncated at 230 base pairs (bp) and reverse reads at 200 bp, no ambiguous bases allowed, and each read required to have <2 expected errors based on their quality scores. Amplicon sequence variants (ASVs) were independently inferred from the forward and reverse reads of each sample using the run-specific error rates. Reads were dereplicated, pairs were merged, and chimeras were removed. Taxonomic assignment was performed against the SILVA v128 database (Pruesse et al., 2007; Quast et al., 2012) using the implementation of the RDP (ribosomal database project) naive Bayesian classifier. A total of 6,715,429 reads (corresponding to ±30,114 reads/samples) were assigned against 20,583 ASVs.

### 16S rRNA qPCR

Quantitative real-time PCR (qPCR) was performed on a Bio-Rad CFX96 thermal cycler to quantify copy number of the bacterial 16S rRNA gene (Suzuki et al., 2000). PCR reactions were performed using the QuantiNova SYBR Green PCR kit (Qiagen) and 0.3 μM of universal bacterial 16S rRNA gene primers 1369F and 1492R (detailed protocol in Supplementary Methods). The results were normalised by the mass of soil or the volume of snow or FT filtered.

### Statistical Analysis

All statistical analyses and visualisations were performed in the R environment using primarily a combination of the vegan (Dixon, 2003), phyloseq (McMurdie and Holmes, 2013) and ggplot2 (Wickham, 2016) packages. The decontam package (Davis et al., 2018) was used to identify potential contaminants using the prevalence function. The ASV table was also manually curated to discard ASVs present in the kit and MiSeq controls in higher abundance than in other samples, leaving 19,081 ASVs. The rarefaction curves saturated, suggesting that we reached the diversity plateau (Supplementary Figure S2). Bacterial richness and diversity indices (Kim et al., 2017) were calculated in phyloseq. Differences in 16S rRNA gene abundance and alpha diversity between sample type (snow, soil, FT), experiment (melt rate or pH), treatment (sterile water or snow) and sampling day and the interaction of all factors were assessed by a multi-factorial design using ANOVA and Tukey’s Honest Significant Difference (HSD) tests with Bonferroni correction.

The ASV table was normalised to the relative abundance and used to evaluate changes in community composition. PERMANOVA were conducted using the adonis function with 999 permutations to identify significant differences in bacterial composition using the Bray–Curtis community dissimilarity and further observed using principal coordinate analysis (PCoA; Ramette, 2007; Paliy and Shankar, 2016). Betadisper (vegan) with 999 permutations was used to test whether the investigated groups were homogeneously dispersed.

To evaluate the colonisation potential, the ASVs identified in the control microcosms and pre-treated soils [day of field sampling (DS), set-up day (D-10) and first day of the experiment (D0)] were discarded from the treated ASV tables. Then, the ASV tables were filtered to keep the ASVs only identified in the snow (541 unique ASVs). Finally, only ASVs identified in the snow and present in the soils after the start of the experiment were conserved to obtain ASV tables of invaders and potential colonists for each experiment, leaving only 16 ASVs as invaders. All other 525 ASVs were either identified only in the FT or snow samples. Invaders included all taxa which were identified in the snow and soil but not detected in the soil prior to the start of the experiment. Potential colonists were invaders that were identified across multiple days throughout the experiments, regardless of whether they were identified in multiple replicates or not. As new members of a community can naturally undergo abundance fluctuations (van Elsas et al., 2012; Acosta et al., 2015; Mallon et al., 2018), ASVs identified across multiple days and still identified on D29 were considered potentially successful colonists, regardless of whether they had increasing or decreasing relative abundances. Differences between the number of invaders, colonists and potentially successful colonists by experiment were tested using ANOVA and Tukey’s HSD tests with Bonferroni correction.

## Results

### The Influence of Melt Rate

Overall, the differences in gene copy numbers were significant between sample types (Table 1). Soil and flow-through (FT) samples had significantly more gene copies than snow samples (Table 1; Supplementary Figure S3A). On average, 4.02 × 10^1^ 16S rRNA gene copies were measured per ml of melted snow and significantly changed by sampling day (Table 1; Supplementary Figure S3A). In soils, the number of gene copies per g of soil decreased during storage but increased again with the start of the experiment and the addition of water. The number of gene copies was higher in the fast flow than in the average flow experiments and the number of gene copies was consistently higher in the controls than in the treated soils (Table 1; Supplementary Figure S3A). On day 29, all samples returned close to the starting number of gene copies (D0), potentially indicating the stabilisation of the soil community 15 days after the end of the melt. In the flow-through samples, higher gene copies were observed in the fast flow than in the average flow experiments (Table 1; Supplementary Figure S3A). In contrast with the soil, the number of gene copies in the flow-through was higher in the treated than in the control samples. Although results were not significant, this is consistent with the addition of microorganisms from the melted snow. In all FT samples, the number of gene copies was highest on day 5 and decreased sharply on all other days. This spike on day 5 was expected as it may reflect the removal of unattached microorganisms, dead cells and relic DNA from the soil, evacuated with the addition of water.

**Table 1:** Results of the ANOVA tests on gene copy numbers (qPCR), richness and Shannon diversity and results of the adonis tests on Bray–Curtis community dissimilarity).

Experiment	Sample type	Formula	Gene copies (qPCR)			Richness			Shannon			Community composition		
			*F*	*p*	Sign	*F*	*p*	Sign	*F*	*p*	Sign	*R* ^2^	*p*	Sign
Melt rate	All	Type	F_2–302_ = 734	2 × 10^−16^	***	F_2–105_ = 72	2 × 10^−16^	***	F_2–105_ = 198	2 × 10^−16^	***	0.36	0.001	***
Melt rate	Snow	Day	F_4–9_ = 15.2	0.0005	***	F_4–5_ = 3.24	0.11		F_4–5_ = 0.96	0.50		0.55	0.008	**
Melt rate	Soil	Rate	F_2–180_ = 11.6	1.9 × 10^−5^	***	F_4 − 103_ = 9.24	2 × 10^−6^	***	F_3 − 103_ = 11.5	9 × 10^−8^	***	0.24	0.001	***
Melt rate	Soil	Treat	F_1–180_ = 6	0.003	**	F_4–103_ = 4.89	0.001	**	F_4 − 103_ = 11.4	1 × 10^−7^	***	0.13	0.004	**
Melt rate	Soil	Rate* Treat	F_1–178_ = 1.89	0.17		F_1–101_ = 0.015	0.90		F_1–101_ = 0.03	0.87		0.02	0.072	
Melt rate	Soil	Rate*Day	F_4–170_ = 2.82	0.03	*	F_6–90_ = 0.66	0.69		F_6–90_ = 0.89	0.51		0.06	0.11	
Melt rate	Soil	Treat *Day	F_4–170_ = 1	0.40		F_6–90_ = 0.39	0.89		F_6–90_ = 0.69	0.66		0.05	0.97	
Melt rate	Soil	Rate* Treat*Day	F_4–160_ = 0.63	0.64		F_4–80_ = 0.25	0.91		F_4–80_ = 0.016	1		0.04	0.67	
Melt rate	FT	Rate	F_1–106_ = 5.53	0.02	*	F_4 − 103_ = 9.24	2 × 10^−6^	***	F_4 − 103_ = 11.5	2 × 10^−8^	***	0.05	0.005	**
Melt rate	FT	Treat	F_1–106_ = 0.69	0.41		F_4–103_ = 4.88	0.001	**	F_4 − 103_ = 11.4	2 × 10^−7^	***	0.03	0.41	
Melt rate	FT	Rate* Treat	F_1–104_ = 0.18	0.67		F_1–101_ = 0.015	0.90		F_1–101_ = 0.03	0.87		0.03	0.25	
Melt rate	FT	Rate*Day	F_2–102_ = 10.9	4.9 × 10^−5^	***	F_6–90_ = 0.66	0.69		F_6–90_ = 0.89	0.51		0.08	0.005	**
Melt rate	FT	Treat*Day	F_2–102_ = 1.58	0.21		F_6–90_ = 0.39	0.89		F_6–90_ = 0.69	0.66		0.05	0.66	
Melt rate	FT	Rate* Treat*Day	F_2–96_ = 0.91	0.41		F_4–80_ = 0.25	0.91		F_4–80_ = 0.02	1		0.05	0.40	
Soil pH	All	Type	F_2–472_ = 161	2 × 10^−16^	***	F_2–165_ = 14.3	2 × 10^−6^	***	F_2–165_ = 125.6	2 × 10^−16^	***	0.36	0.001	***
Soil pH	Snow	Day	F_4–8_ = 25.3	0.0001	***	F_4–5_ = 0.86	0.55		F_4–5_ = 0.55	0.71		0.49	0.051	
Soil pH	Soil	pH	F_3–296_ = 152	2 × 10^−16^	***	F_3–101_ = 19.02	7.5 × 10^−10^	***	F_3–101_ = 22	5 × 10^−11^	***	0.32	0.001	***
Soil pH	Soil	Treat	F_2–296_ = 1.61	0.20		F_2–102_ = 2.13	0.12		F_2–102_ = 2.96	0.06		0.03	0.17	
Soil pH	Soil	pH* Treat	F_2–293_ = 0.27	0.77		F_2–98_ = 5.15	0.0075	**	F_2–98_ = 6.6	0.002	**	0.04	0.005	**
Soil pH	Soil	pH*Day	F_10–280_ = 3.19	0.0007	***	F_9–85_ = 4.84	3.2 × 10^−5^	***	F_9–85_ = 3.4	0.001	**	0.13	0.001	***
Soil pH	Soil	Treat*Day	F_5–286_ = 0.44	0.082		F_5–90_ = 0.80	0.55		F_5–90_ = 0.39	0.86		0.53	1	
Soil pH	Soil	pH* Treat*Day	F_10–262_ = 2.27	0.015	*	F_9–68_ = 0.99	0.46		F_9–68_ = 0.68	0.73		0.05	0.53	
Soil pH	FT	pH	F_2–159_ = 6.21	0.003	**	F_2–50_ = 1.56	0.22		F_2–50_ = 0.88	0.42		0.07	0.001	***
Soil pH	FT	Treat	F_1–160_ = 0.32	0.57		F_1–51_ = 0.11	0.74		F_1–51_ = 1.67	0.20		0.02	0.70	
Soil pH	FT	pH*Treat	F_4–156_ = 0.30	0.75		F_2–47_ = 0.5	0.61		F_2–47_ = 0.11	0.90		0.03	0.79	
Soil pH	FT	pH*Day	F_4–153_ = 7.91	7 × 10^−6^	***	F_4–44_ = 1.23	0.31		F_4–44_ = 1.23	0.30		0.09	0.003	**
Soil pH	FT	Treat*Day	F_4–144_ = 0.36	0.70		F_2–47_ = 0.96	0.39		F_2–47_ = 2.98	0.06		0.04	0.55	
Soil pH	FT	pH* Treat*Day	F_4–144_ = 0.35	0.84		F_4–35_ = 2.06	0.11		F_4–35_ = 5.12	0.002	**	0.08	0.13	

The experiment column refers to either the different melt rate or soil pH microcosms. The sample type column differentiates between snow, soil and flow-through (FT). The formula column indicates the parameters used in the models. Type = sample type/rate = fast or slow/treatment (treat) = snow or sterile water input/day = day of sampling. Value of *p*, asterisk indicates significant differences (Sign).

* *p* < 0.05;

** *p* < 0.01;

*** *p* < 0.001.

**Figure 2:**
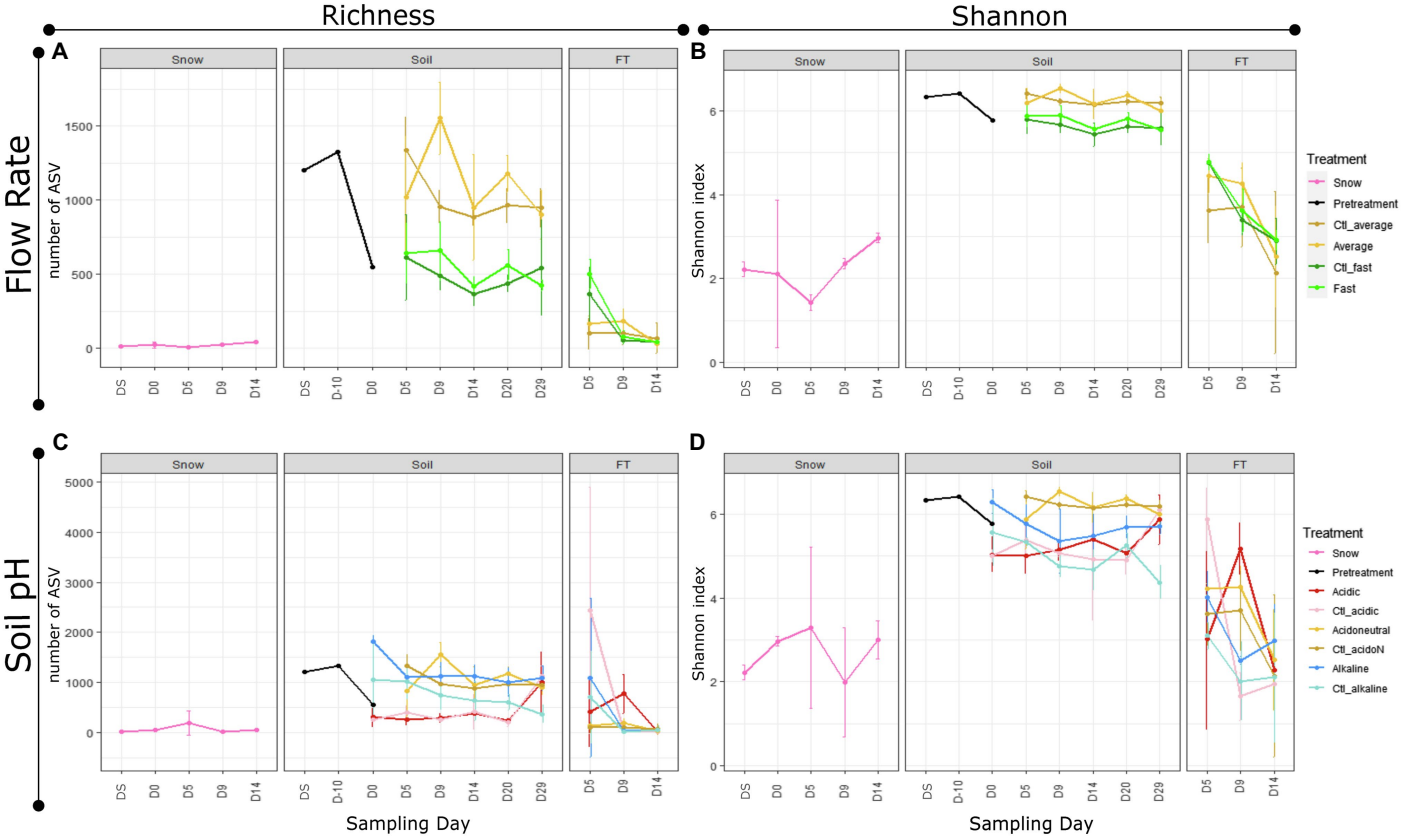
Alpha diversity by experiment, treatment, sample type and day of sampling. **(A)** Observed number of amplicon sequence variants (ASVs) (Richness) and **(B)** diversity with the Shannon index in different flow rate experiments. **(C)** Observed number of ASVs (Richness) and **(D)** diversity with the Shannon index in different soil pH experiments. The shades of yellow correspond to the average and acidoneutral controls (Ctl) and treatments, resulting from the same microcosms.

Differences in alpha diversity were significant by sample type (Figures 2A,B; Table 1). In the snow, differences from the day of field sampling until the end of the experiments were not significant (Figures 2A,B; Table 1). In the soils, as with gene copies, there was a decrease in alpha diversity measures with storage (pre-treated), which increased at the beginning of the experiment with the addition of water (Figures 2A,B; Table 1). Alpha diversity was significantly higher in the average flow rate. However, there was no significant difference between control and treated samples and the day of sampling had limited influence on soil alpha diversity measures (Figures 2A,B; Table 1). In the flow-through, the number of ASVs was significantly higher in the fast flow experiments but decreased with time, highlighting the spike at D5, also observed with gene copy numbers (Figures 2A,B; Table 1).

**Figure 3:**
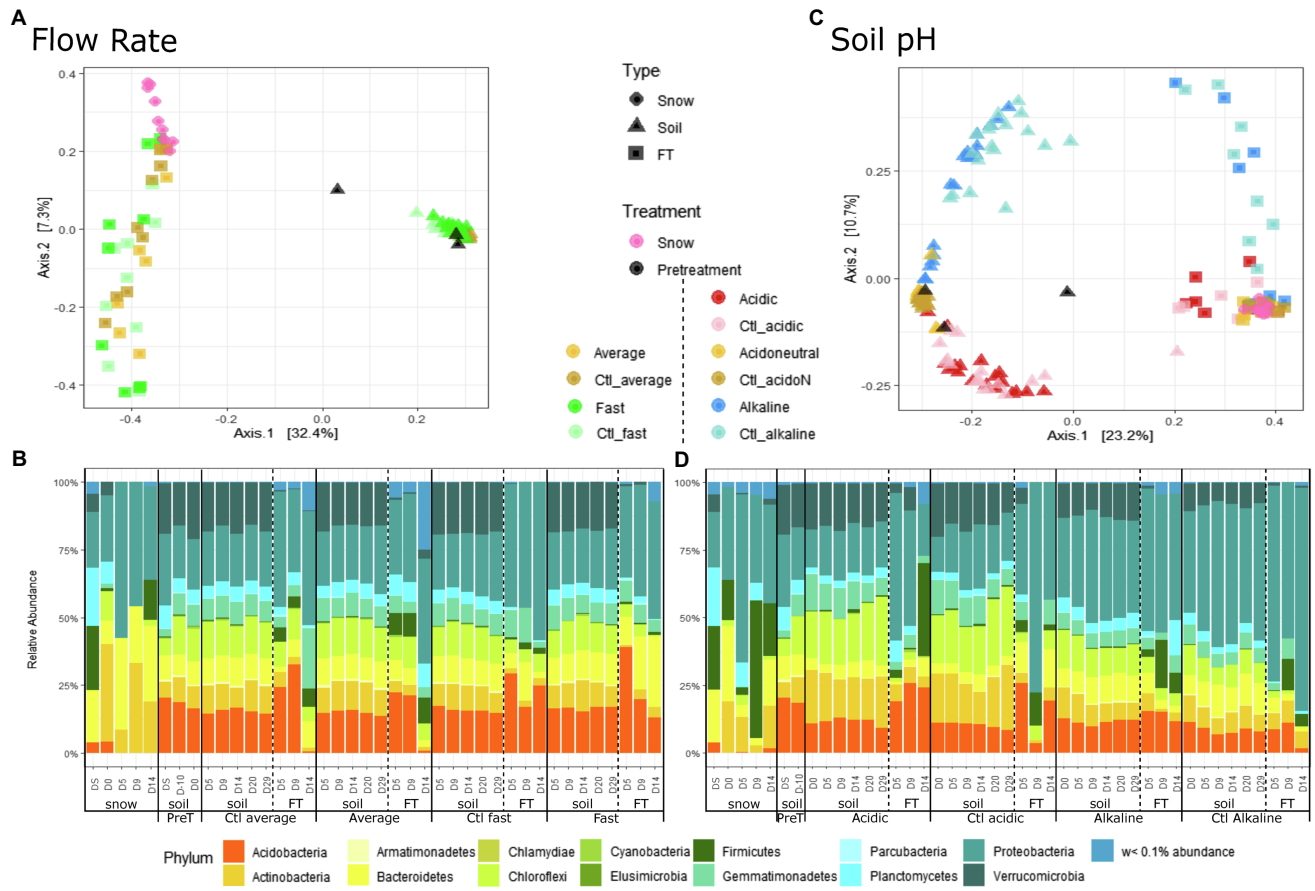
Principal coordinate analysis (PCoA) and community composition at the phylum level, by experiment, treatment, sample type and day of sampling. **(A)** PCoA and **(B)** community composition of flow rate microcosms. **(C)** PCoA and **(D)** community composition of soil pH microcosms. Pretreatment (PreT) includes the day of field sampling (DS), the day of microcosm set-up (D-10) and D0 for the flow rate experiment as the soil was not yet treated in any way. ‘w<0.1% abundance’ regroups all phyla with less than 0.1% relative abundance.

Overall, differences in bacterial community composition by sample type were observed using PCoA based on the Bray–Curtis dissimilarity (Figure 3A; Table 1), although these differences were likely also influenced by within-sample dispersion (betadisper *F* = 151, *p* < 0.001). Snow communities changed significantly between the day of field sampling and the end of the experiment (Table 1) and were primarily composed of Proteobacteria and Bacteroidetes (Figure 3B; Supplementary Figure S4A). Large variations in the composition of FT samples masked the potential variation in soil samples (Figure 3A) and therefore, the PCoA of soil communities was assessed separately (Supplementary Figure S5). The soil community on the day of field sampling changed with storage (Figure 3A; Supplementary Figure S5). Soil communities clustered separately between flow rates, but as for alpha diversity, controls and treated samples were similar (Table 1; Supplementary Figure S5). We should note that group dispersion was heterogeneous (betadisper *F* = 7.4, *p* < 0.001). Soil communities were primarily composed of Acidobacteria, Actinobacteria, Bacteroidetes, Chloroflexi, Gemmatimonadetes, Planctomycetes, Proteobacteria and Verrucomicrobia (Figure 3B; Supplementary Figure S4A). The communities identified in the flow-through presented clear variations, primarily separated by flow rate and sampling day (Figure 3). All FT samples were dominated by Proteobacteria but differences in communities with flow rate were observed. For instance, all fast FT samples had large proportions of Acidobacteria, Bacteroidetes and Proteobacteria and were relatively stable while in the average rate, communities significantly changed on D14 with the depletion of Acidobacteria (Figure 3B; Supplementary Figure S4A).

### The Influence of Soil pH

Here, we assessed the influence of soil pH on the colonisation potential of snow microorganisms. The results from the average flow rate were used as the acidoneutral samples for comparison. As in the melt rate experiments, the differences in gene copy numbers were significant between sample types, with soil and FT samples harbouring significantly more gene copies than snow samples, which changed significantly by sampling day (Table 1; Supplementary Figure S3B). In soils, the number of gene copies decreased during storage between the day of field sampling (DS) and D-10. After pH manipulation (D-10) and until D0, the number of gene copies increased in alkaline soils but decreased in acidic soils. During the experiment, the gene copy number remained lowest in acidic and highest in alkaline soils with no significant differences between controls and treated soils (Table 1; Supplementary Figure S3B). In the flow-through, higher gene copies were quantified in the alkaline soils, which also presented the highest variability (Supplementary Figure S3B; Table 1). As observed in the melt rate experiments, the number of gene copies peaked on D5 and decreased with time.

Differences in alpha diversity were significant by sample type (Figures 2C,D; Table 1). In the snow, alpha diversity did not change significantly from the day of sampling until the end of the experiments (Figures 2C,D; Table 1). In the soils, as with gene copies, there was a decrease in alpha diversity measures with storage (pre-treated). However, alpha diversity changed following pH manipulation showing an increase in alkaline soils and a decrease in acidic soils (Figures 2A,B). During the experiment, both soil pH and snow/water treatment were identified as significant variables influenced by the day of sampling (Table 1). Interestingly, on day 29, all microcosms harboured similar alpha diversity levels, except the alkaline controls (Figures 2C,D), suggesting stabilisation of the communities. In the flow-through, alpha diversity decreased with time and the interactions between soil pH, treatment and sampling day were significant (Figures 2C,D; Table 1).

Overall, differences in bacterial community composition by sample type were observed [groups were homogeneously dispersed (betadisper *F* = 0.93, *p* = 0.40)] (Figure 3C; Table 1). In the PCoA, the snow samples clustered closely together, indicating similar communities over time (Figure 3C; Table 1) dominated by Proteobacteria and Firmicutes (Supplementary Figure S4B). Acidic, acidoneutral and alkaline soil samples all formed different clusters indicating important differences in community composition (Figure 3C). The horseshoe effect observed in the PCoA likely reflects the pH gradient and highlights the change in community composition across this gradient (Morton et al., 2017), although these differences were likely also influenced by within-sample dispersion (betadisper *F* = 8.40, *p* < 0.001). This difference in soil community composition was further observed in Figure 3D, illustrating the community composition at the phylum level. We observed a shift in community composition during storage but also after pH manipulation, between D-10 and D0. Acidic communities presented an increase in Actinobacteria and Chloroflexi while an increase in Proteobacteria was observed in alkaline communities (Supplementary Figure S4B). The addition of water after the start of the experiments further enhanced this increase in Proteobacteria (Figure 3D; Supplementary Figure S4B). Acidic soil communities were dominated by Actinobacteria, Chloroflexi, Proteobacteria and Actinobacteria while Alkaline soil communities were dominated by Proteobacteria (Figure 3D; Supplementary Figure S4B). Flow-through samples we dominated by Proteobacteria Supplementary Figure S4B) and displayed high variability in community composition (Figures 3C,D; Table 1).

### Colonisation Potential

**Figure 4:**
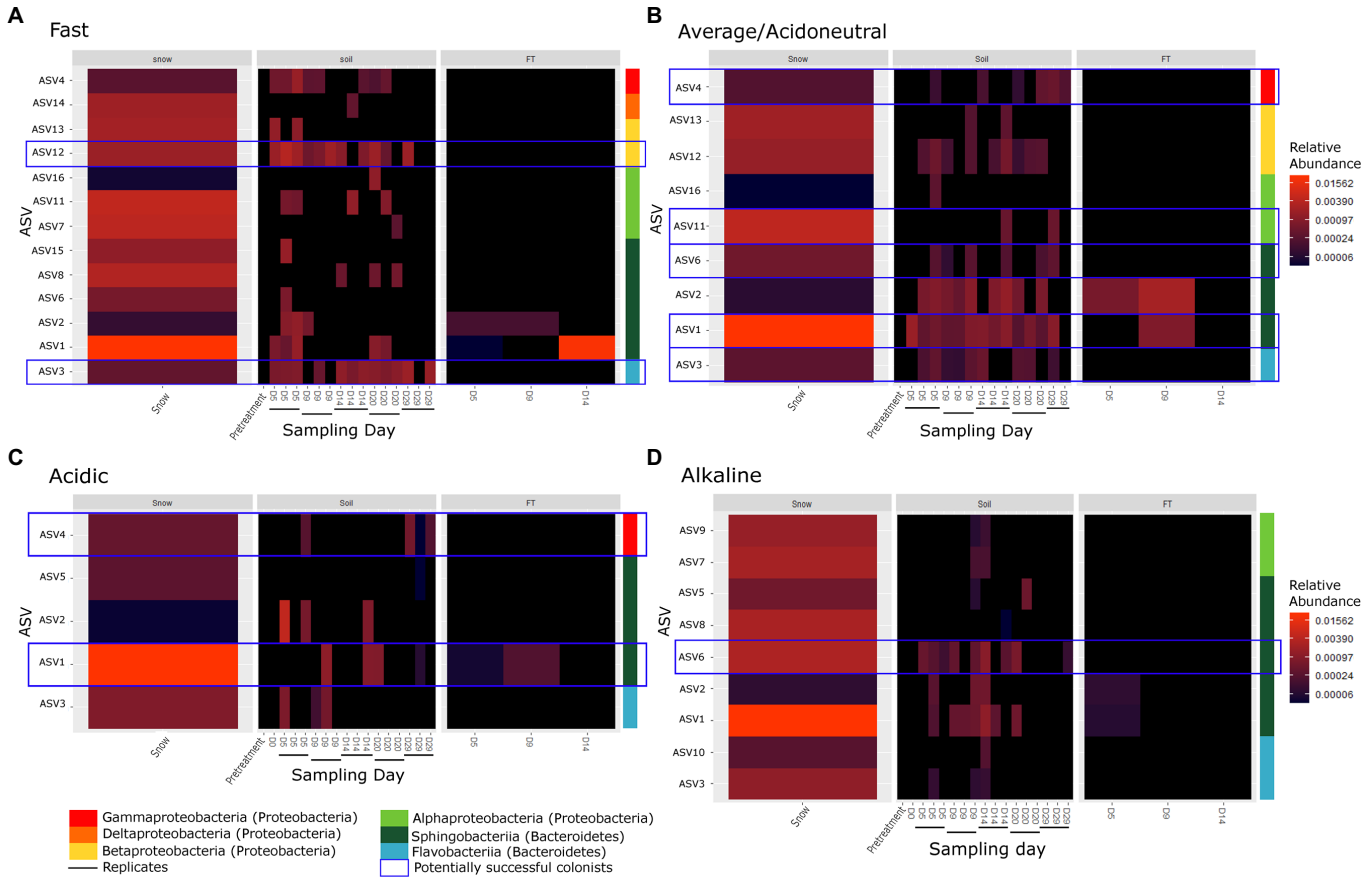
Heatmap of invaders of **(A)** the fast flow, **(B)** the average flow/acidoneutral, **(C)** the acidic, and **(D)** the alkaline experiments where each row corresponds to an ASV. The coloured bar indicates the taxonomy associated to each ASV at the class level. Blue boxes highlight potentially successful colonists identified across multiple days and still present on D29. Black horizontal bars indicate replicate microcosms.

To assess whether colonisation of the soil by microorganisms in the snow occurred, only the ASVs identified in the snow, absent from the controls and pre-treated soils (DS, D-10 and D0) but identified in the soils from D5 were selected for further analysis. Only 16 ASVs fulfilled these conditions and were considered potential invaders. All were classified as Proteobacteria or Bacteroidetes, primarily Alphaproteobacteria, Betaproteobacteria, Flavobacteriia and Sphingobacteriia at the class level (Figure 4; Supplementary Table S2). In some cases, we identified taxa on D20 or D29 that were not previously identified and appeared new. It is likely that these taxa were previously present but had abundances below the detection threshold.

In the fast flow experiments, a total of 13 ASVs were identified as invaders depositing in the soils (Figure 4A; Supplementary Table S2). Of these 13 invaders, seven were identified across multiple days and were considered potential colonists. However, only two persisted until D29 suggesting potentially successful colonists. In the average flow experiments (acidoneutral experiments), nine ASVs were identified as invaders (Figure 4B; Supplementary Table S2). Of these nine ASVs, eight were identified across multiple days and considered potential colonists. Five ASVs persisted until D29 and were considered potentially successful colonists. Differences in invaders, colonists and potentially successful colonists were not statistically significant between flow rates. In acidic soils, only five ASVs were identified as invaders (Figure 4C; Supplementary Table S2). Of these five invaders, four were identified across multiple days and considered potential colonists; however, only two persisted until D29. In alkaline soils, nine ASVs were identified as invaders (Figure 4D). Of these nine invaders, seven were identified across multiple days and considered potential colonists but only one persisted until D29. Overall, the persistence of taxa until the end of the experiment was highest at the average flow in acidoneutral soils, with five potentially successful colonisation events. The higher flow rate increased the number of invaders but the persistence across time decreased. In acidic soils at the average flow rate, the number of invaders and persistence with time was much lower. In alkaline soils, the number of invaders was equal to that observed in acidoneutral soils but persistence with time was strongly reduced. Statistically, the number of invaders and colonists was significantly higher in acidoneutral soils (ANOVA, *p* = 0.02 and *p* = 0.009 respectively), and potentially successful colonists were only marginally more successful (ANOVA, *p* = 0.056).

## Discussion

In this study, we assessed the influence of increased precipitation (*via* the melt rate) and soil pH on the colonisation potential of snow microorganisms. Overall, we rejected the first hypothesis that increased precipitation (higher flow rate) would promote successful colonisations due to the higher inoculum density as well as increased ecosystem disturbance. However, we did support the second hypothesis by observing increased colonisation in acidoneutral soils compared to acidic and alkaline soils.

In both sets of experiments, the number of gene copies was consistently higher in the control than in the treated soils. This may be a signal of invasion, that is, species in the controls were not experiencing interactions with outside invaders, did not have to partition resources and could therefore grow to higher abundances. This signal of invasion was further observed for the richness and diversity, consistently higher in the treated soils, in line with the addition of snow microorganisms.

Of the two flow rate scenarios tested, the fast flow rate increased the input of microorganisms to the system, as would be expected from increased precipitation, and was expected to cause greater disturbance than the average melt rate. Ecosystem disturbance is considered a key factor for successful colonisation by newly deposited microbial invaders, with increasing disturbance enhancing the chances of successful colonisation (Liu et al., 2012; De Roy et al., 2013). We expected the addition of water at the higher flow rate to disturb soil communities (observed *via* changes in alpha and beta diversity) and increase the colonisation potential. Instead, the strongest impact on communities was observed at the average rate. Richness and diversity increased, and the soil community composition shifted away from the composition on the first day of the experiment (Supplementary Figure S5). Furthermore, melted snow has been shown to add nutrients to the ecosystem, resulting in a nutrient pulse of carbon, nitrogen and phosphorus (Schmidt and Lipson, 2004; Edwards et al., 2006; Buckeridge and Grogan, 2010; Larose et al., 2013). As nutrient pulses increase the chances of successful colonisation (Li and Stevens, 2012; Van Nevel et al., 2013; Mallon et al., 2015b), we expected to observe differences between the controls and treated samples. Instead, controls and treated soils within each experiment remained similar and only differences between experiments (average and fast rates) were observed. The shift in communities between average and fast flow rate suggested physical selection of microorganisms. For instance, fast growing r-strategists or EPS (extracellular polymeric substance) producing bacteria might be selected in the fast flow microcosms to ensure attachment, aggregation and growth in the soil system (Ho et al., 2017; Costa et al., 2018). Overall, following the increased input of microorganisms, we observed more invaders but low persistence with time and only two potentially successful colonists. At the average rate, the number of invaders was lower but persistence with time increased and five potentially successful colonists were identified, suggesting that a slower rate may give more time for invading microorganisms to compete with indigenous microorganisms and successfully colonise the ecosystem instead of being pushed out of the system by a higher flow rate.

As pH has previously been identified as a key driver of bacterial community structure in global (Fierer and Jackson, 2006; Delgado-Baquerizo et al., 2018) and in Arctic soils (Malard et al., 2019a), and dispersal is an important process structuring bacterial communities, the influence of soil pH on the colonisation success was investigated. The soil pH appeared to have a stronger influence on soil bacterial communities than flow rate. Not only were richness and diversity lower than in acidoneutral soils, but community composition was clearly different, forming three distinct clusters in the PCoA. Contrary to the flow rate experiments, communities between control and treated soils were also different, showing that the input of snow or sterile water had different consequences, potentially due to the input of nutrients from the snow and the different bacterial communities in acidic and alkaline soils. Acidic soils contained the lowest number of invaders deposited, as only two were considered potentially successful colonists. In alkaline soils, as in acidoneutral soils, an equal number of invaders were identified. However, persistence with time was much lower and only one potentially successful colonist was identified. The low colonisation observed in acidic and alkaline samples and successful colonisation in acidoneutral soils supports the second hypothesis that acidoneutral soils would promote successful colonisations. Furthermore, it suggests that while the flow rate is an important parameter in promoting successful colonisation, soil pH may be an even more important factor limiting colonisation.

Overall, all potentially successful colonists were identified in multiple replicates across different time points within each experiment, as well as in different experiments (Figure 4; Supplementary Table S2), suggesting active and selective colonisation of the soil by these taxa. For instance, ASV3 is a colonist at both flow rates, ASV1 and 4 are colonists of both acidoneutral and acidic soils while ASV6 colonised acidoneutral and alkaline soils. The potentially successful colonists (Proteobacteria and Bacteroidetes) were primarily classified as r-strategists (as in Fierer et al., 2007 and Ho et al., 2017), with high growth rates and generally considered more likely to successfully colonise (Andrews and Harris, 1986; Litchman, 2010).

The colonisation and persistence with time observed in acidoneutral soils was in agreement with the consensus that acidoneutral soils may decrease the adaptative pressure on microbial communities (Voroney and Heck, 2014; Morris and Blackwood, 2015). Arctic acidoneutral soils harbour more generalist taxa (Malard et al., 2019a), generally considered more prone to dispersal (Pandit et al., 2009; Graham and Stegen, 2017; Sriswasdi et al., 2017). Therefore, the higher successful colonisation of acidoneutral soils further supported the role of dispersal in shaping bacterial communities in these ecosystems.

On the other hand, the lower number of invading taxa in acidic soils was not surprising considering that most bacterial taxa decrease in abundance with decreasing pH (Chu et al., 2010; Rousk et al., 2010), including the invading taxa Bacteroidetes. Potentially successful colonists of acidic and alkaline soils colonised acidoneutral soils, however, they did not colonise each other. The low rate of colonisation of acidic and alkaline soils, both generally characterised as harsh (Fierer and Jackson, 2006; Rousk et al., 2010), may highlight the need for effective adaptations (Beales, 2004; Ward et al., 2009) lacking in the deposited microorganisms. They may also be worse competitors than the indigenous soil communities, generally dominated by specialist taxa (Malard et al., 2019a) and acclimated to the soil pH. However, in this study, we opted to use the same soil bacterial community as a baseline to evaluate the colonisation potential. Therefore, we manipulated the soil pH by the addition of aluminium sulphate and calcium carbonate (Nicol et al., 2008). While we let the communities acclimatise to the new soil pH, they may be less adapted than indigenous communities in naturally acidic and alkaline soils. The addition of sulphate coupled with the addition of water could have formed sulphuric acid, especially at lower flow rates where the soil was exposed to air. Toxic metals, such as manganese, aluminium and arsenic, may have been released (Robson and Abbott, 1989; Kunhikrishnan et al., 2016). On the other hand, calcium carbonate is used to increase soil pH and reduce the possible toxicity of manganese and aluminium ions (Muhlbachova and Tlustos, 2006; Kunhikrishnan et al., 2016; Guo et al., 2019). In all manipulated pH microcosms, we observed a shift in bacterial communities following these adjustments (between D-10 and D0). Future studies could repeat these experiments using soils within the targeted pH range and the associated indigenous communities to establish if the colonisation potential is also lower in these natural systems. It is also interesting to note that the snow pH was acidoneutral (6.33 ± 0.15) and therefore, snow microorganisms may have already been well adapted for acidoneutral soil conditions. As snow pH is generally acidoneutral (Kol and Taylor, 1942; Drake and Moore, 1980; Newton, 1982; Li et al., 2007; Ali et al., 2010; Zhang et al., 2013), this scenario is rather realistic. However, we cannot exclude the possibility that acidic or alkaline precipitation may promote colonisation in soils within the same pH range.

Overall, local environmental conditions may be more important determinant factors influencing the outcome of colonisation than increased precipitation or faster melt events. Furthermore, we did not observe the inverse relationship between microbial diversity and invasion outcome previously identified or theorised (van Elsas et al., 2012; Mallon et al., 2015a; Kinnunen et al., 2016; Vila et al., 2019). Here, more potential colonisation events were observed in the […]. Instead, only invaders presenting the right adaptations to colonise a free niche (Malard et al., 2021) or outcompete indigenous microorganisms may be able to successfully colonise in the medium to long term. Following the deposition, the invaders have to adapt, compete for resources and then grow and spread in the ecosystem to colonise (Mallon et al., 2015a; Kinnunen et al., 2016). Here, we demonstrated that microorganisms were successfully deposited but only few taxa had the potential to successfully colonise the soils. After 15 days, the majority had disappeared leading to likely failed colonisations; although, even failed colonisations may influence indigenous microbial communities (Mallon et al., 2018).

## Conclusion

This study used the Arctic snowpack as a model system to investigate microbial colonisation of snow bacteria deposited into Arctic soils. First, we tested the impact of increased precipitation (inoculum density) and the subsequent faster snowmelt rate (ecosystem disturbance) on the colonisation outcome. We identified more invaders but decreased persistence with time and a lower number of successful colonisation events. We also evaluated the influence on soil pH on this colonisation potential. Here, persistence with time decreased and a lower number of successful colonisation events were recorded in acidic and alkaline soils compared with acidoneutral soils. Overall, we demonstrated that soil bacterial communities could change significantly with snowmelt and that microorganisms were successfully deposited in soils following snowmelt events. However, we showed that only few taxa successfully colonised and established in these soil communities. Results suggest that local soil properties might have a greater influence on the colonisation outcome than increased precipitation or ecosystem disturbance.

## Data Availability Statement

The datasets presented in this study can be found in online repositories. The names of the repository/repositories and accession number(s) can be found at: https://www.ncbi.nlm.nih.gov/, PRJNA564428.

## Author Contributions

DP secured the funding. LM and DP conceived and designed the study. LM carried the experimental work, laboratory work, bioinformatics processing, statistical analysis, and drafted the manuscript. DP revised and approved the final version. All authors contributed to the article and approved the submitted version.

## Funding

This work was supported by a grant from the European Commission’s Marie Sklowdowska Curie Actions program under project number 675546.

## Conflict of Interest

The authors declare that the research was conducted in the absence of any commercial or financial relationships that could be construed as a potential conflict of interest.

## Publisher’s Note

All claims expressed in this article are solely those of the authors and do not necessarily represent those of their affiliated organizations, or those of the publisher, the editors and the reviewers. Any product that may be evaluated in this article, or claim that may be made by its manufacturer, is not guaranteed or endorsed by the publisher.
