# Supplementary material for: Bacterial Colonisation: From Airborne Dispersal to Integration Within the Soil Community
Source: Front Microbiol. 2022 May 9;13:782789. doi: 10.3389/fmicb.2022.782789 (PMC9125085; doi:10.3389/fmicb.2022.782789)
Supplement: Supplementary file 1 [file Data_Sheet_1.docx]

**Supplementary information**

***Material and Methods***

1. *Microcosms construction*

The sterile water used in the controls consisted of filtered MilliQ water added to a clean and empty autoclaved container and autoclaved again. The sterility of the water was assessed on each sampling day by microscopy using Petroff-Hausser chambers and DNA extractions. 10 µL of undiluted sterile water was input in the chamber and the number of cells was counted. This process was repeated 4 times at each sampling event and the numbers were found to be consistently below the detection level (<1 cell observed/10µL). The sterility of the water was further confirmed by DNA extraction and qPCR which yielded concentrations too low to amplify.

1. *16S rRNA qPCR*

Quantitative real-time PCR (qPCR) was performed on a CFX96 thermal cycler (Bio-Rad) to quantify copy number of the bacterial 16S rRNA gene in samples of interest. All preparations were carried in a Scientific Optimiser PCR Workstation (CBS), and the required equipment was exposed to UV light. PCR reactions were performed using the QuantiNova SYBR Green PCR kit (Qiagen), universal bacterial 16S rRNA gene primers 1369F (5’-CGGTGAATACGTT CYCGG-3’) and 1492R (5’-GGWTACCTTGTTACGACTT’), initially described by Suzuki et al. (2000). The manufacturer’s protocol was followed, except for the primer concentration which was set at 0.3 µM. This primer concentration decreased the primer dimer formation and increased the reaction specificity. Reactions were set up in 96 well, white-walled PCR assay plates (Bio-Rad) and covered with clear heat seal (Bio-Rad) prior to measurements. The total volume of each reaction was 20 µL, composed of 10 µL of SYBR Green PCR Master Mix, 0.6 µL of each primer and variable quantities of DNA and PCR grade water. Soil samples were characterised by a high biomass and therefore, 1 µL of DNA was sufficient for quantification, in addition to 7.8 µL of water. Snow and liquid samples had low biomass, therefore, 5 µL of DNA and 3.8 µL of water was added to each reaction. The standard curve was based on samples of known genomic DNA content from *Escherichia coli* K12. Samples were loaded on the thermal cycler and run at 95° C for 2 min, followed by 40 cycles at 95° C for 5 s and 56° C for 10 s. The build-in melt curve analysis was carried after the 40 cycles and consisted of a gradual increase in temperature from 65° C to 95° C. The success of each qPCR run was evaluated by the qPCR efficiency between 90 % and 110 %, along with an R2 >0.98. Then, a linear regression calculated the 16S rRNA gene copy number in experimental samples. The melt curve analysis was used to assess the specificity of the primers, where a single peak is expected. In all high biomass sample, this condition was respected, illustrating the specificity of the reaction. However, in low biomass samples, a second peak was often identified. It was visually compared with the ‘no template control’ (NTC), composed on the SYBR green master mix and primers. The melting peak of the NTC illustrated the primer dimer peak, which corresponded to the second peak observed in low biomass samples. It confirmed the good specificity of the reaction with primer dimer formation in the case of low biomass samples. After quantification, the results were normalised by the volume of input DNA, the volume of DNA extracted and the volume/mass of the initial sample.

**Figures**

**Figure S1**: Snow, water and soil pH changes with time. Each point indicates the mean pH measured. We observe the overall conservation of pH within the initial pH category all along the experiment.


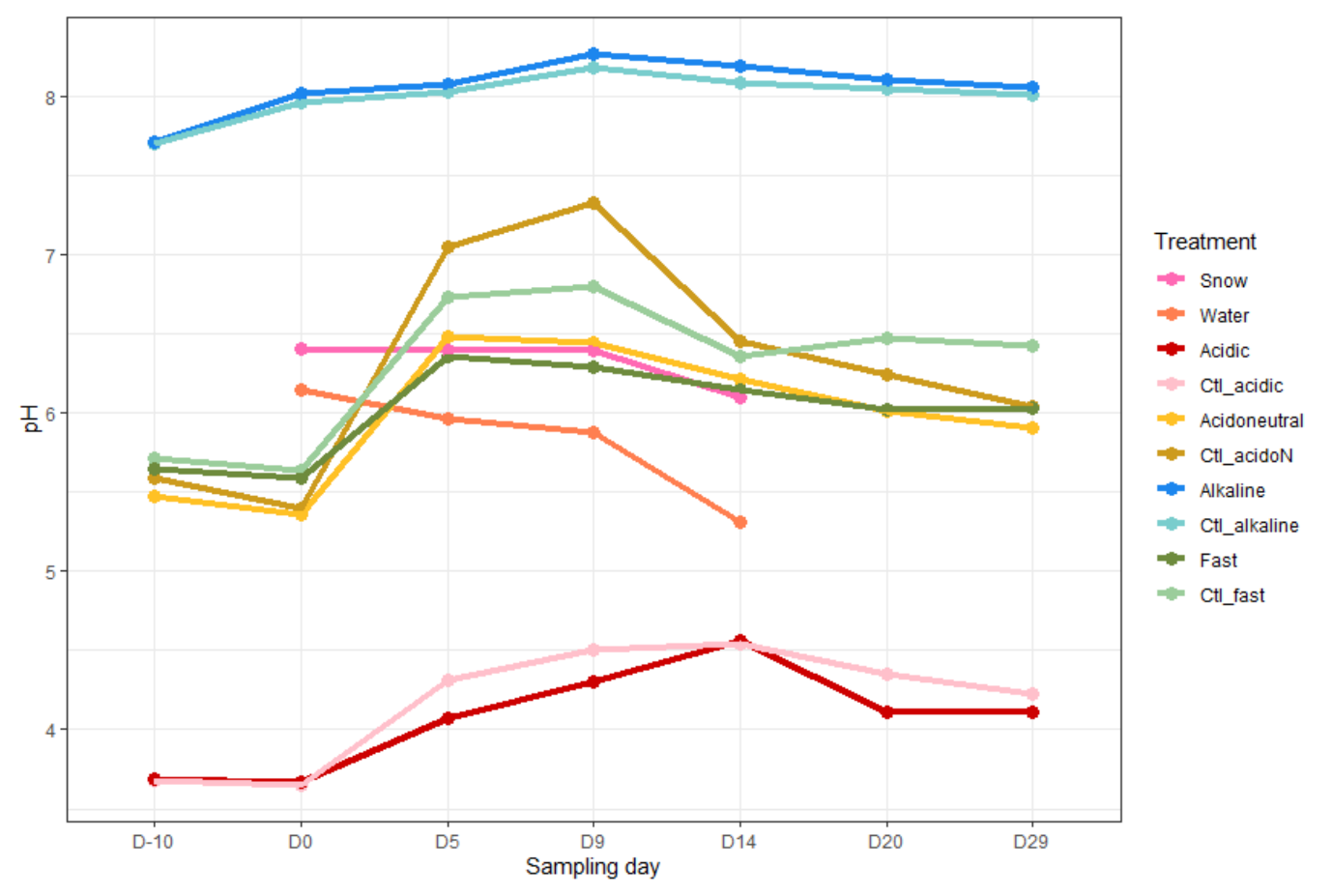


**Figure S2**: Bacterial rarefaction curves by sample type for A. flow rate and B. soil pH experiments. The levels of diversity were similar and asymptotic for both sets of experiments.


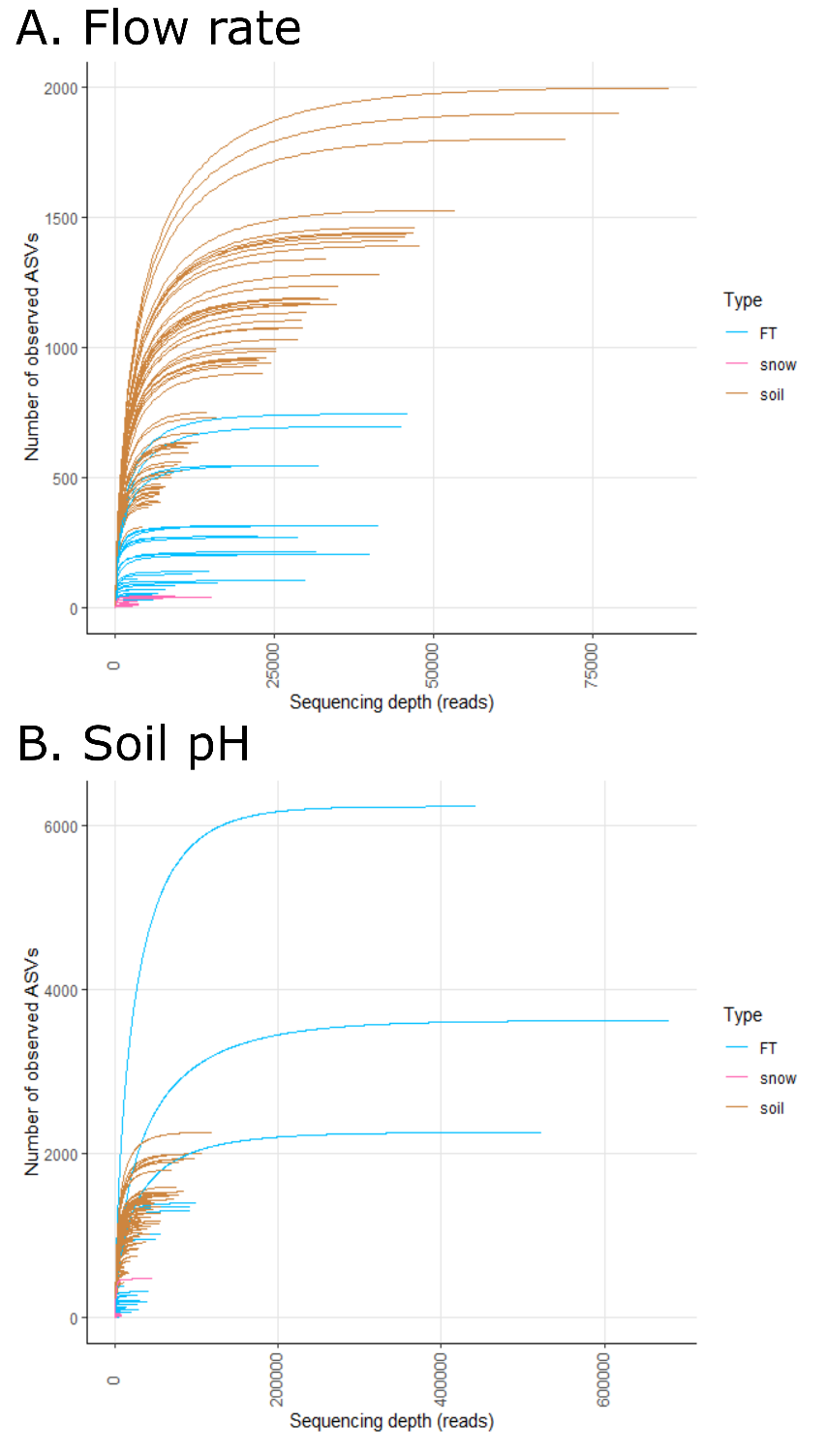


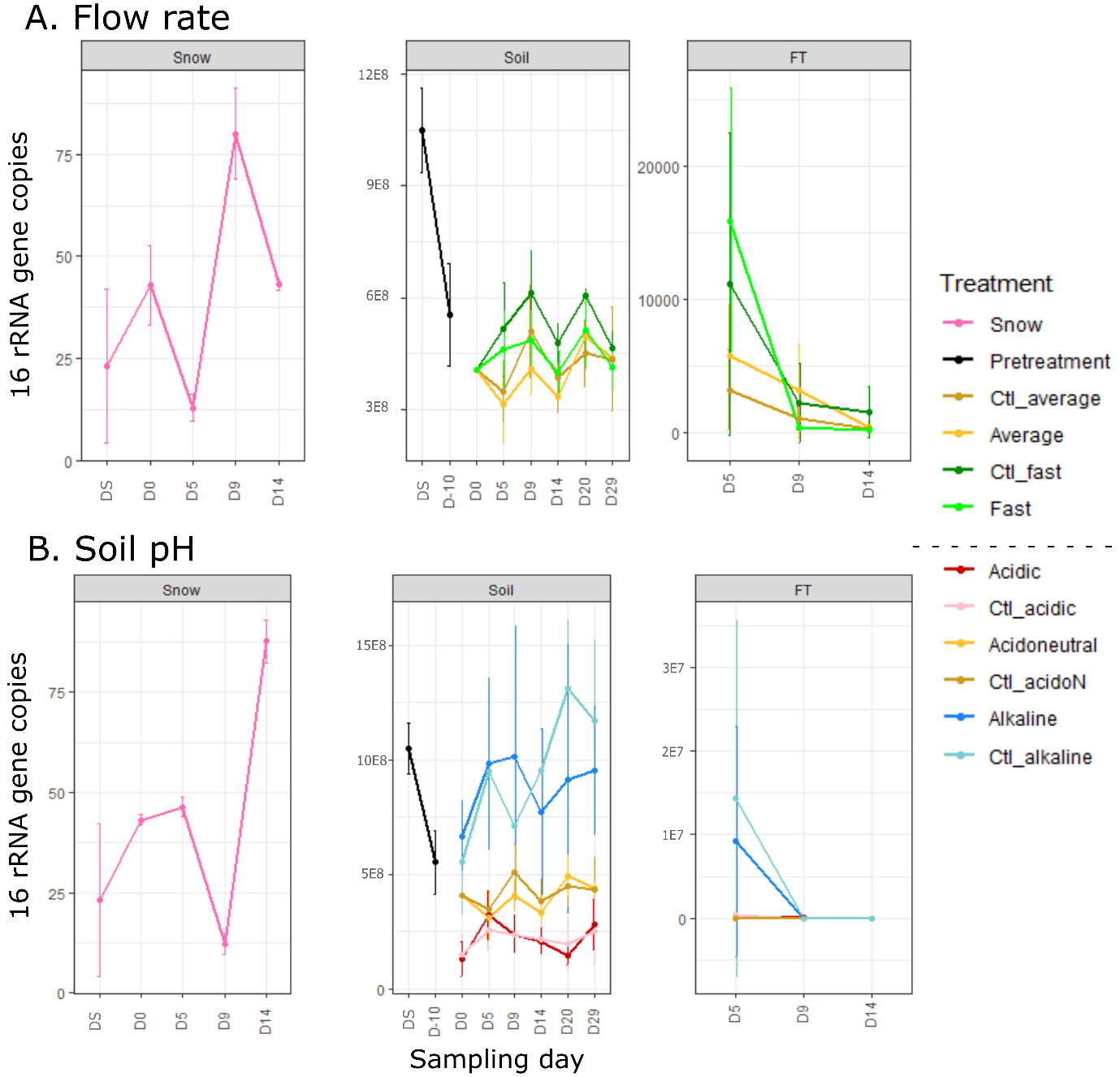


**Figure S3**: Normalised abundance of 16S rRNA gene copies per mL of melted snow, per mL of FT and per g of soil, as measured by qPCR.

**
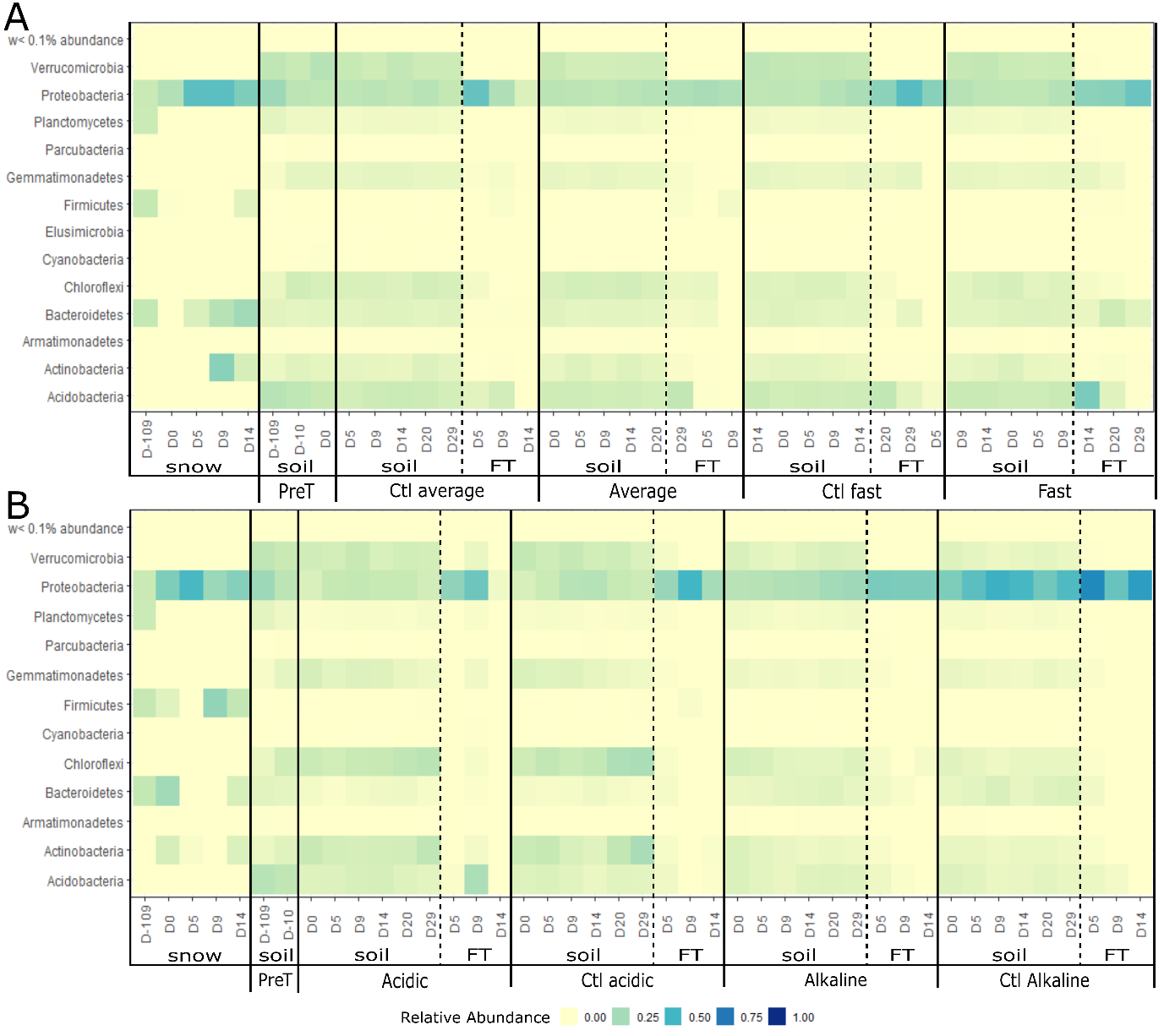
Figure S4:** Heatmap illustrating the relative abundance of the dominant phyla in each experiment. Pretreatment (PreT) includes the day of field sampling (DS), the day of microcosm set-up (D-10) and D0 for the flow rate experiment as the soil was not yet treated in any way.


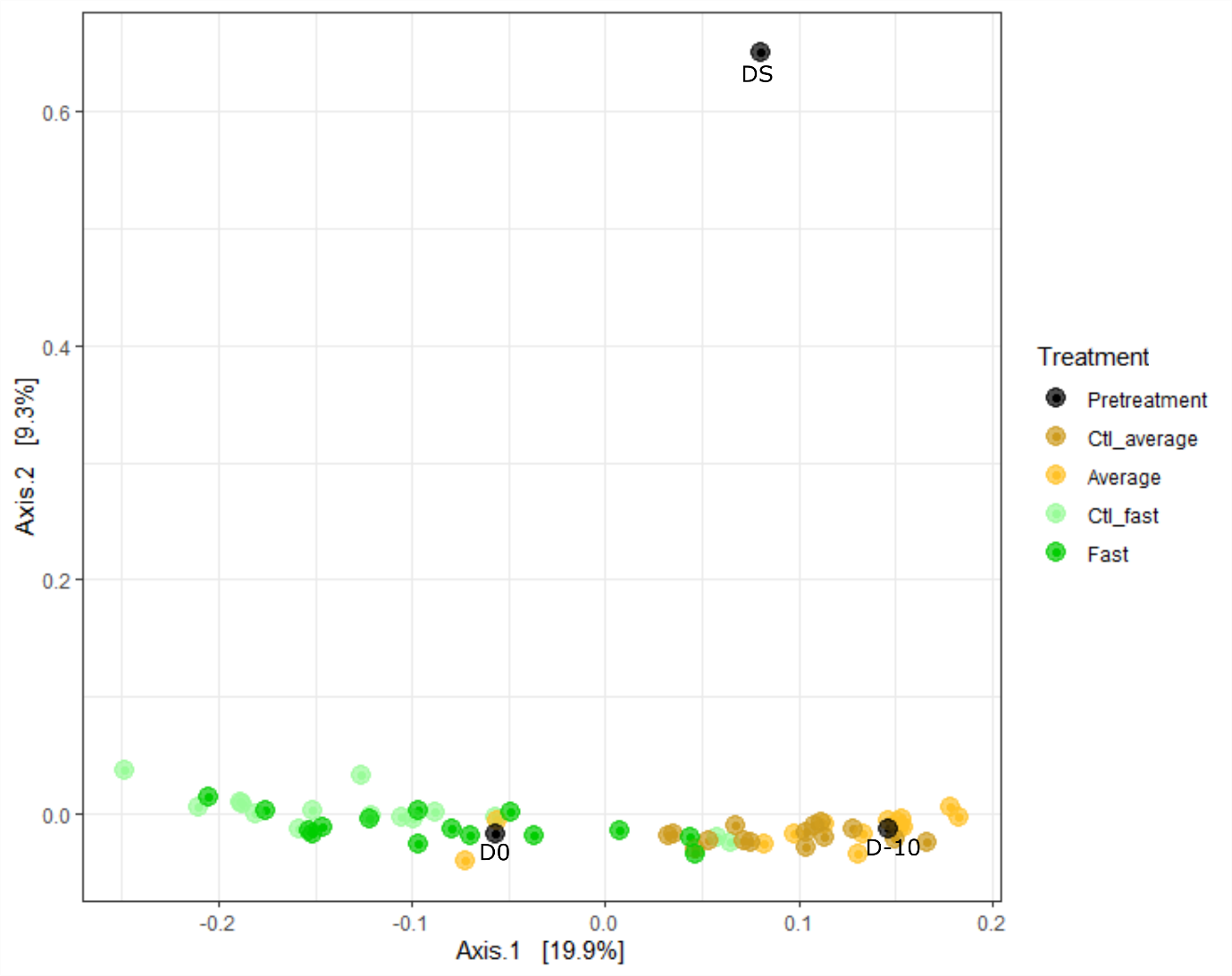


**Figure S5**: PCoA of soil microbial communities of the flow rate experiment illustrating the difference in communities with flow rates as well as changes in community composition with storage. The pre-treatment communities correspond to samples before any treatment started. DS indicates the day of field sampling while D-10 is ten day before the beginning of the experiment and D0 is the day of the experiment.

**Tables**

**Table S1:** Soil properties prior to any manipulations

|  | TOC (%) | Moisture (%) | pH | Conductivity (µS) |
| --- | --- | --- | --- | --- |
| Soil | 7.6 | 17.59 | 5.31 | 109 |

<0.01; *,

**Table S2:** Classification at the deepest taxonomic level of all the invaders and potential colonists identified in this study. Kingdom, Phylum, Class, Order, Family.

| **ASV** | **Taxonomy** | **Colonist** |
| --- | --- | --- |
| ASV1 | Bacteria, Bacteroidetes, Sphingobacteriia, Sphingobacteriales, Chitinophagaceae | Yes |
| ASV2 | Bacteria, Bacteroidetes, Sphingobacteriia, Sphingobacteriales, Sphingobacteriaceae | No |
| ASV3 | Bacteria, Bacteroidetes, Flavobacteriia, Flavobacteriales, Flavobacteriaceae | Yes |
| ASV4 | Bacteria, Proteobacteria, Gammaproteobacteria, Xanthomonadales, Nevskiaceae | Yes |
| ASV5 | Bacteria, Bacteroidetes, Flavobacteriia, Flavobacteriales, Flavobacteriaceae | No |
| ASV6 | Bacteria, Proteobacteria, Deltaproteobacteria, Myxococcales | Yes |
| ASV7 | Bacteria, Proteobacteria, Alphaproteobacteria, Rhodospirillales, Acetobacteraceae | No |
| ASV8 | Bacteria, Bacteroidetes, Sphingobacteriia, Sphingobacteriales | No |
| ASV9 | Bacteria, Proteobacteria, Alphaproteobacteria, Sphingomonadales, Sphingomonadaceae | No |
| ASV10 | Bacteria, Bacteroidetes, Flavobacteriia, Flavobacteriales, Flavobacteriaceae | No |
| ASV11 | Bacteria, Proteobacteria, Alphaproteobacteria, Sphingomonadales, Sphingomonadaceae | Yes |
| ASV12 | Bacteria, Proteobacteria, Betaproteobacteria, Burkholderiales, Oxalobacteraceae | Yes |
| ASV13 | Bacteria, Proteobacteria, Betaproteobacteria, Burkholderiales, Oxalobacteraceae | No |
| ASV14 | Bacteria, Proteobacteria, Deltaproteobacteria, Myxococcales | No |
| ASV15 | Bacteria, Bacteroidetes, Sphingobacteriia, Sphingobacteriales, Sphingobacteriaceae | No |
| ASV16 | Bacteria, Proteobacteria, Alphaproteobacteria, Sphingomonadales, Sphingomonadaceae | No |

**References**

Suzuki, M.T., Taylor, L.T., DeLong, E.F.J.A.E.M., 2000. Quantitative analysis of small-subunit rRNA genes in mixed microbial populations via 5′-nuclease assays. 66, 4605-4614.
